# EphA2-specific microvesicles derived from tumor cells facilitate the targeted delivery of chemotherapeutic drugs for osteosarcoma therapy

**DOI:** 10.1186/s12951-024-02372-0

**Published:** 2024-03-03

**Authors:** Zhenggang Wang, Zhiyi He, Junlai Wan, Anmin Chen, Peng Cheng, Wentao Zhu

**Affiliations:** 1 Department of Orthopedics, Tongji Hospital, Tongji Medical College, Huazhong University of Science and Technology, Wuhan, 430030 China; 2 Division of Spine Surgery, Department of Orthopedic Surgery, Nanjing Drum Tower Hospital, Affiliated Hospital of Medical School, Nanjing University, Nanjing, 210008 China

**Keywords:** EphA2, Osteosarcoma, Nanoplatform, Surface functionalization, Microvesicles, Tumor targeting

## Abstract

Despite advances in surgery and chemotherapy, the survival of patients with osteosarcoma (OS) has not been fundamentally improved over the last two decades. Microvesicles (MVs) have a high cargo-loading capacity and are emerging as a promising drug delivery nanoplatform. The aim of this study was to develop MVs as specifically designed vehicles to enable OS-specific targeting and efficient treatment of OS. Herein, we designed and constructed a nanoplatform (YSA-SPION-MV/MTX) consisting of methotrexate (MTX)-loaded MVs coated with surface-carboxyl Fe3O4 superparamagnetic nanoparticles (SPIONs) conjugated with ephrin alpha 2 (EphA2)-targeted peptides (YSAYPDSVPMMS, YSA). YSA-SPION-MV/MTX showed an effective targeting effect on OS cells, which was depended on the binding of the YSA peptide to EphA2. In the orthotopic OS mouse model, YSA-SPION-MV/MTX effectively delivered drugs to tumor sites with specific targeting, resulting in superior anti-tumor activity compared to MTX or MV/MTX. And YSA-SPION-MV/MTX also reduced the side effects of high-dose MTX. Taken together, this strategy opens up a new avenue for OS therapy. And we expect this MV-based therapy to serve as a promising platform for the next generation of precision cancer nanomedicines.

**Graphical Abstract:**

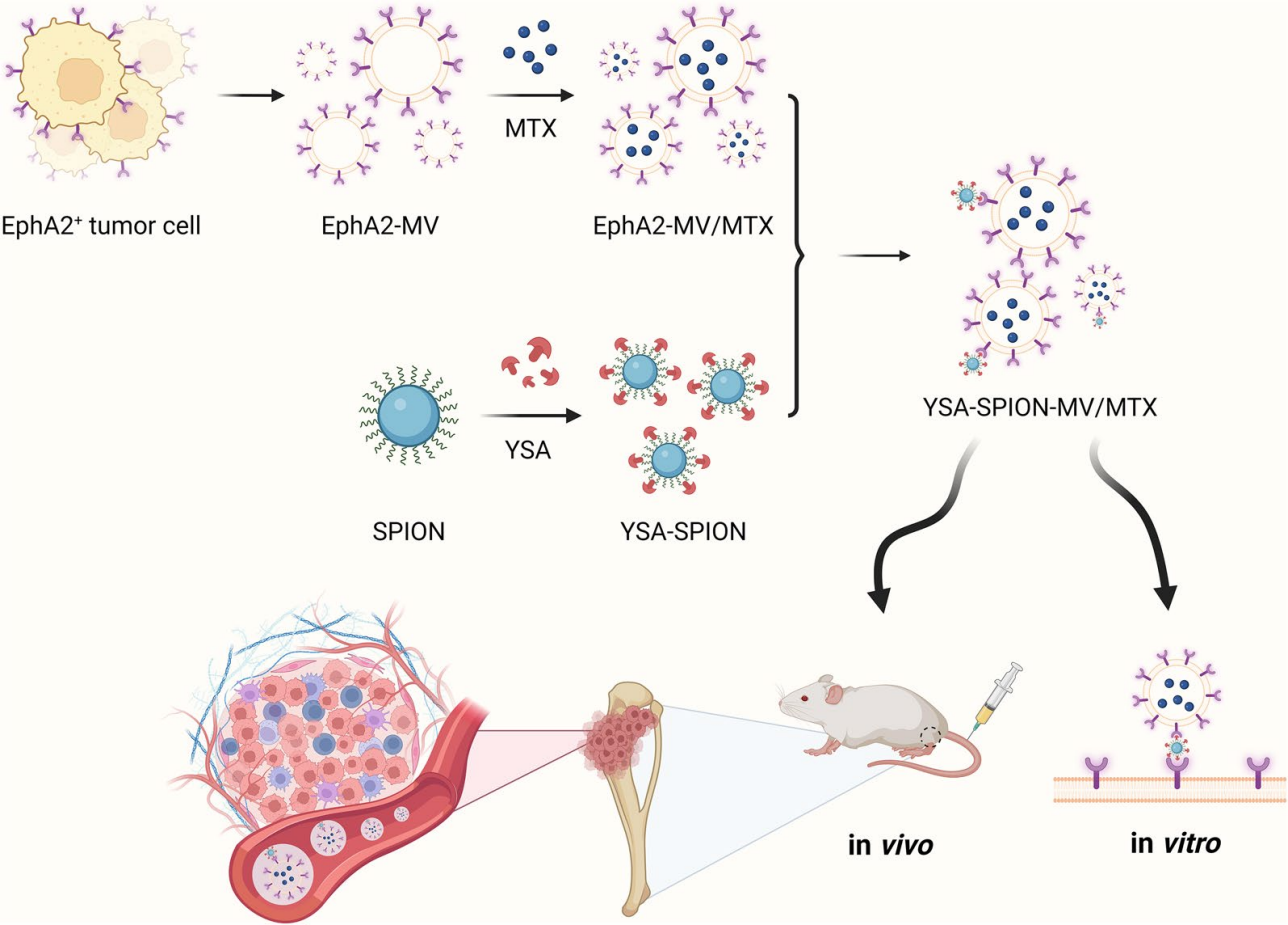

**Supplementary Information:**

The online version contains supplementary material available at 10.1186/s12951-024-02372-0.

## Background

Osteosarcoma (OS), the most common primary malignant bone tumor in children and adolescents, severely affects patients' limb function and quality of life [1, 2]. Distant metastases and pathological fractures caused by bone destruction are the most common complications [3]. Despite the incorporation of surgery and chemotherapy, the survival of OS patients has not been fundamentally improved over the last two decades, mainly due to lung micrometastases and chemotherapy resistance [4, 5]. Although methotrexate is the main drug used in chemotherapy regimens, side effects caused by high-dose systemic administration are a major challenge in clinical management [6]. Therefore, innovative and effective drug delivery platforms are urgently needed.

Nanoparticle-based drug delivery systems have been extensively explored in recent years. Microvesicles (MVs) are natural lipid bilayer vesicles with a diameter of 50–1000 nm which are secreted by cells, belonging to a subtype of extracellular vesicles [7, 8]. In comparison to conventional nanoparticles, MVs exhibit low toxicity, high biocompatibility and low immunogenicity [9]. In addition, MVs possess the following advantages: (1) The lipid bilayer structure can protect their contents from degradation, enhance in vivo stability, and prolong blood circulation time; (2) MVs inherit specific receptors from their parent cells, providing certain inherent targeting capabilities; (3) MVs have a certain permeability across biological barriers [9–11]. However, most natural MVs for systemic administration cannot be effectively enriched at the site of disease, especially in organs that are protected by biological barriers [12, 13].

Over the past several years, click chemistry has been utilized to affix targeting peptides to the surface of MVs, thus improving their ability to be effectively delivered to specific tissues or cells [14, 15]. Previous studies have shown that ephrin alpha 2 receptor (EphA2), a member of the Eph family of receptor tyrosine kinases, is highly expressed in osteosarcoma lines and primary osteosarcoma cells, but not in normal bone cells [16]. YSA (YSAYPDSVPMMS) is a bioactive ephrin mimetic peptide that can selectively bind to EphA2 [17]. Therefore, it can serve as a homing peptide for targeted delivery to osteosarcoma. Superparamagnetic iron oxide nanoparticles (SPIONs), which possess excellent superparamagnetism, good biocompatibility and low toxicity, have a widespread application in biomedicine [18, 19]. With the development of surface chemistry, it is feasible to enhance the binding affinity of SPIONs with MVs by functionalizing them with tailored surface ligands [20].

Hereby, we rationally designed and successfully fabricated the functionalized chemotherapeutic MVs (YSA-SPION-MV/MTX) for targeted osteosarcoma therapy. Briefly, we isolated EphA2-positive microvesicles (EphA2-MV) from the culture medium of EphA2^+^ tumor cells (143B and MG63), and loaded them with methotrexate (EphA2-MV/MTX). Subsequently, we coated surface-carboxyl Fe_3_O_4_ superparamagnetic nanoparticles (SPIONs) with a high-density YSA peptide by coupling reaction (YSA-SPION). Based on the excess YSA peptide on the surface of YSA-SPION, YSA-SPION and EphA2-MV/MTX formed a nanocomposite named as YSA-SPION-MV/MTX. Finally, the targeting ability and anti-tumor activity of this nanocomposite were evaluated in vitro and in vivo using an orthotopic OS mouse model. Overall, this work provides an appealing strategy for targeted therapy of osteosarcoma and other malignancies, and also provides valuable insights into the development of MVs as drug delivery vehicles.

## Results

### Characterization of MVs

**Fig. 1:**
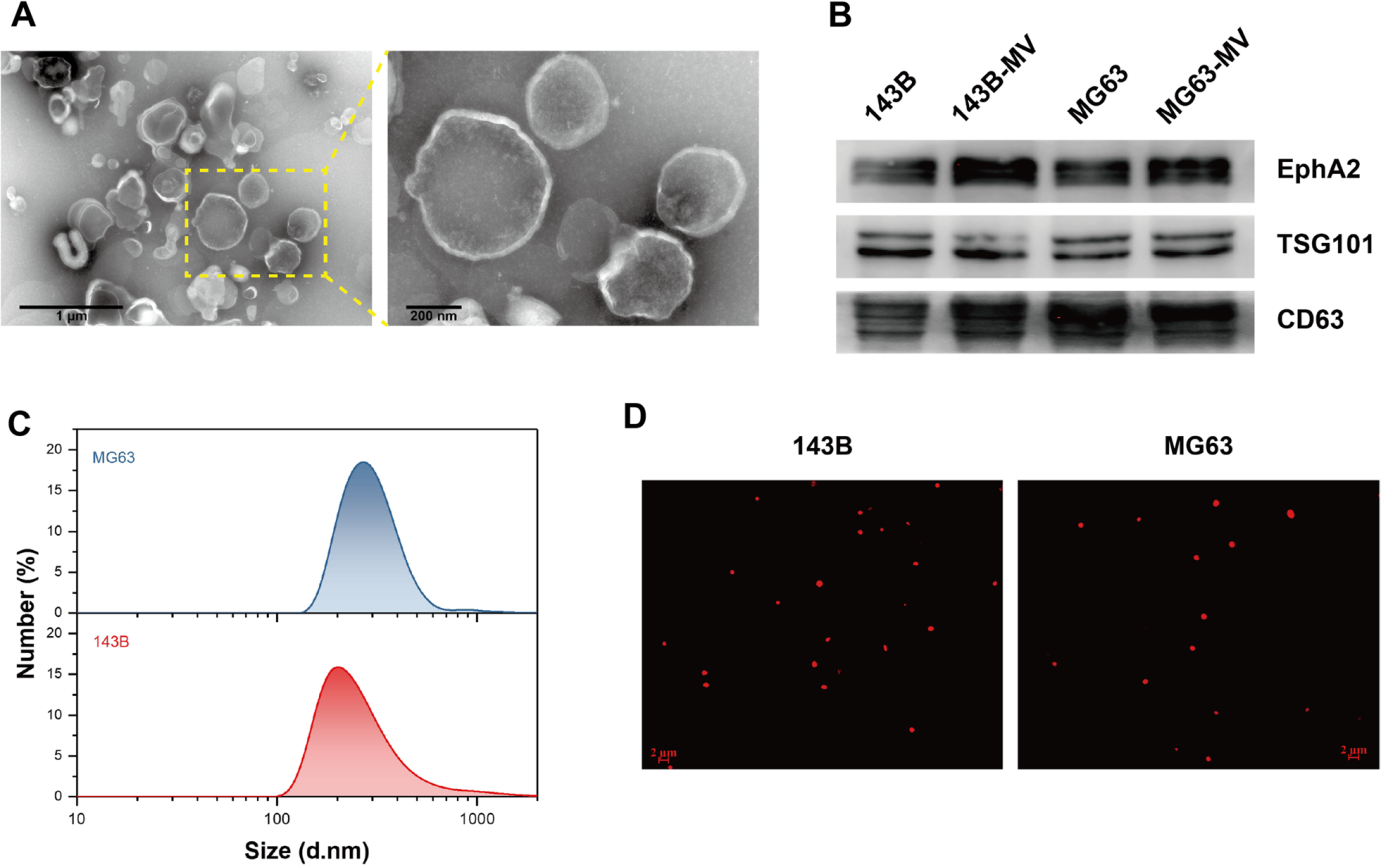
Characterization of MVs. **A** Representative TEM images of MVs (scale bars: 1 μm and 200 nm). **B** Western blot analysis for the expression of EphA2, TSG101 and CD63. **C** Particle size distribution of MVs measured by DLS. **D** MVs were observed under a confocal microscope and DOX was shown as a red signal (scale bars: 2 μm)

As shown in Fig. 1A, round-like MVs with clear membrane structures were observed by TEM. Western blot analysis showed that tumor cell-derived MVs expressed characteristic marker proteins TSG101 and CD63, along with high levels of EphA2 (Fig. 1B). The particle size distribution of MVs ranged from 100 to 1000 nm, with the majority having a diameter of 200–400 nm (Fig. 1C). Using to a flow cytometry-based method, it was shown that 1 × 10^7^ MVs could be isolated from 20 mL supernatant obtained from 7 × 10^7^ tumor cells (Additional file 1: Fig. S1). Given the fluorescent nature of DOX, we used DOX fluorescence to determine whether the drug was packaged into MVs. A red fluorescent signal was clearly observed under a confocal microscope, indicating that DOX was encapsulated by MVs (Fig. 1D). Furthermore, the drug concentration of MV/MTX was approximately 5 μg/1 × 10^7^ MVs by LS-MS/MS analysis (Additional file 2: Fig. S2).

### Characterization of YSA-SPION

**Fig. 2:**
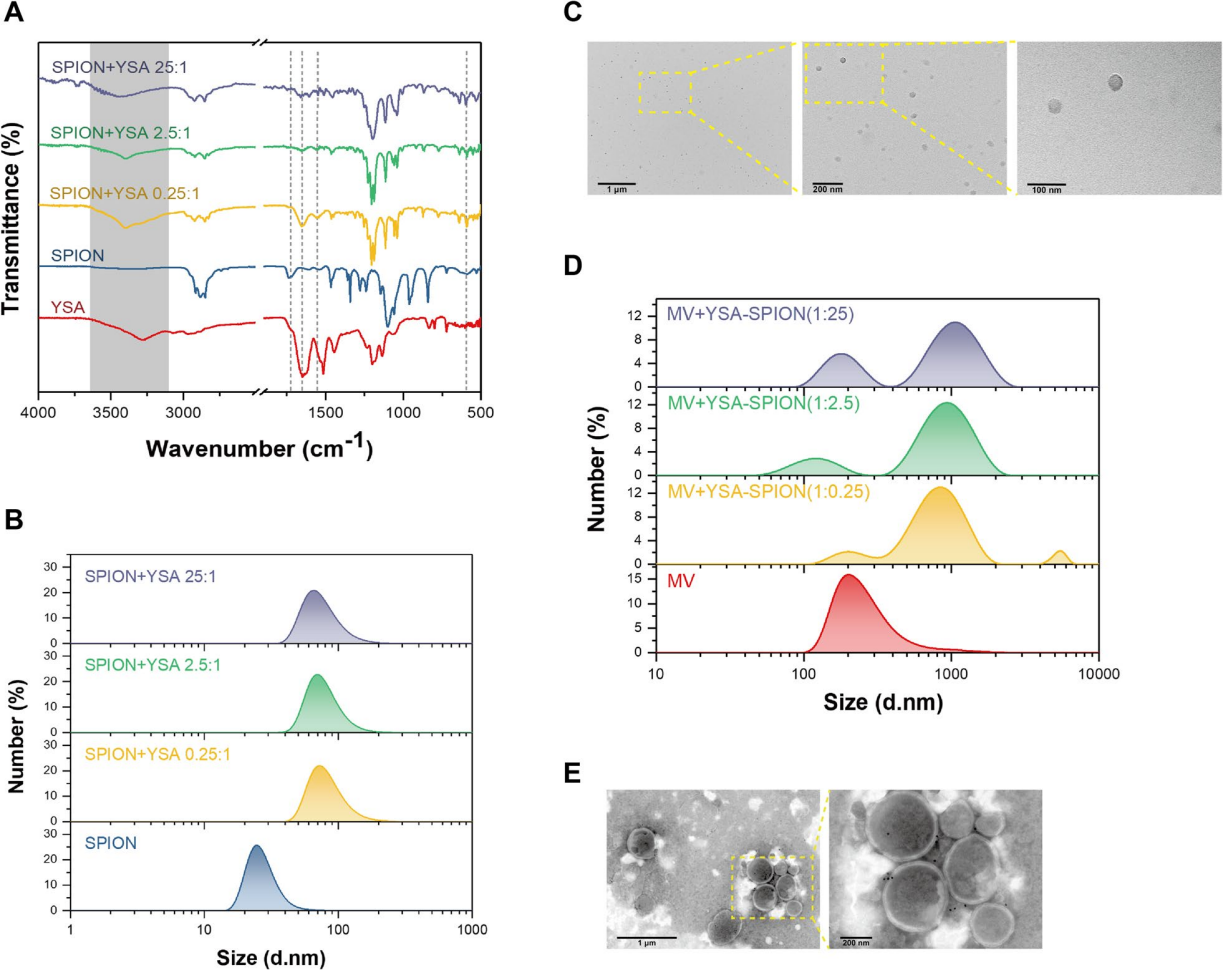
Characterization of YSA-SPION and YSA-SPION-MV. The **A** FT-IR spectrum and **B** particle size distribution of YSA-SPION. **C** Representative TEM images of SPION (scale bars: 1 μm, 200 nm, 100 nm). **D** Particle size distribution of reaction systems with different component ratios. **E** Representative TEM images of YSA-SPION-MV (scale bars: 1 μm, 200 nm)

SPION was bound to YSA peptide at different ratios (SPION:YSA = 25:1, 2.5:1, and 0.25:1) using a coupling reaction (Additional file 3: Fig. S3A). As shown in Fig. 2A, for the FT-IR spectrum of YSA peptide and SPION, the absorption peaks at 586 cm^−1^ belonged to the stretching vibration mode of Fe–O bonds, the absorption band at 3420 cm^−1^ represented the –OH of the terminal carboxyl, and the intense and broad peak at 3100–3500 cm^−1^ was due to the primary amine group or the terminal hydroxyl group in the peptide [21, 22]. For the FT-IR spectrum of YSA-SPION, the absorption of amide carbonyl groups occurred at 1650 cm^−1^; the bending frequency of amide N–H appeared at 1555 cm^−1^ [21]. In addition, 1726 cm^−1^ represented the stretching vibration peak of C=O in the free carboxyl group and disappeared in the YSA-SPION spectrum, indicating the formation of amide bands and ester bands. To sum up, the YSA peptide was successfully coated onto SPION.

The particle size distribution of SPION ranged from 15–60 nm, with the majority having a diameter of 20–40 nm (Fig. 2B, C). After coating with YSA peptide, the diameter of YSA-SPION (YSA:SPION = 1:25, 1:2.5, and 1:0.25) increased to approximately 76 nm, 79 nm and 93 nm, respectively (Fig. 2B).

### Characterization of YSA-SPION-MV

100 μL MV (1 × 10^7^/mL) was mixed with 100 μL YSA-SPION (different proportions) overnight at 4 °C, and a visible deposit formed in the reaction system with YSA-SPION (1:0.25) (Additional file 3: Fig. S3B). As shown in Fig. 2D, after modification by YSA-SPION, the proportion of MVs with a diameter of 200–400 nm decreased, and a new type of particles (YSA-SPION-MV) with a diameter of 600–1000 nm was detected. Correspondingly, particles with a diameter of about 5000 nm were also detected in the reaction system with YSA-SPION (1:0.25). Moreover, TEM showed that YSA-SPION-MV still had a round-like membrane structure, and MVs were surrounded by multiple SPION (Fig. 2E).

### Drug release and stability of YSA-SPION-MV/MTX

As shown in Additional file 4: Fig. S4A, the release of MTX from YSA-SPION-MV/MTX displayed a pH-sensitive pattern. After 48 h, the release rates of MTX reached 73.52% ± 1.67% at pH5.5 and only 34.15% ± 1.45% at pH7.4. This finding suggested that YSA-SPION-MV/MTX might restrict MTX release in the blood circulation, while accelerate MTX release in an acidic tumor microenvironment [23, 24].

As shown in Additional file 4: Fig. S4B, after being stored for 48 h at 4 °C, the relative content of SPION in YSA-SPION-MV/MTX did not change significantly compared to its fresh sample. And after being stored for 2 weeks at − 80 °C, the changes in gross characters (clarity, color, and solubility), particle size and morphology of YSA-SPION-MV/MTX are negligible (Additional file 4: Fig. S4C–E). These results implied that YSA-SPION-MV/MTX had good stability.

### Cell uptake and targeting capability of YSA-SPION-MV in vitro and in vivo

**Fig. 3:**
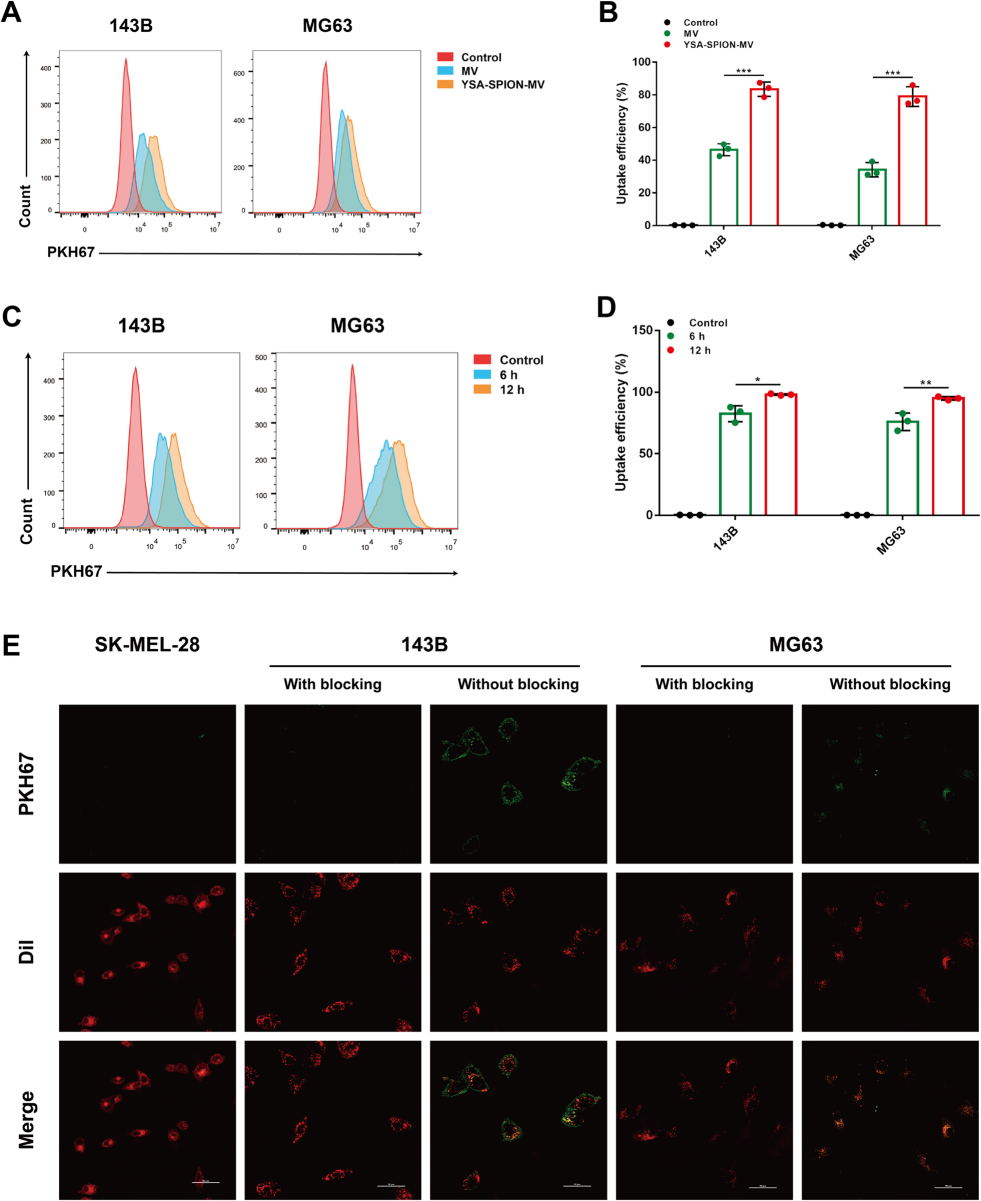
Cell uptake and targeting capability of YSA-SPION-MV in vitro. Flow cytometry analysis and bar graph of PKH67 positive cells: i) incubation with MV or YSA-SPION-MV for 6 h (**A**, **B**); ii) incubation with YSA-SPION-MV for 6 h or 12 h (**C**, **D**). **E** Fluorescence images of PKH67-labeled YSA-SPION-MV treated 143B and MG63 cells with and without blocking by YSA peptide, and SK-MEL-28 cells (scale bars: 50 μm). *P < 0.05; **P < 0.01; ***P < 0.001

Based on the above results, we confirmed that the functionalized chemotherapeutic MVs were successfully synthesized. Prior to treatment, the cell uptake and targeting capability were assessed. After 6-h incubation, flow cytometry analysis showed high uptake of YSA-SPION-MV (143B: 83.47% ± 4.41%; MG63: 79.00% ± 6.04%) compared to MV (143B: 46.33% ± 3.68%, *P* < 0.001; MG63: 34.10% ± 4.56%, *P* < 0.001), indicating that the chemotherapeutic drug could be delivered more effectively by YSA-SPION-MV (Fig. 3A, B). And the uptake increased over the incubation time (143B: 82.43% ± 6.44% *vs.* 98.00% ± 0.66%, *P* = 0.0011; MG63: 75.90% ± 7.02% *vs.* 94.93% ± 1.40%, *P* = 0.0002) (Fig. 3C, D).

EphA2^+^ 143B and MG63 cells were blocked with YSA peptide following treatment with PKH67-labeled YSA-SPION-MV, leading to a decrease in fluorescence intensity of PKH67 when compared to unblocked cells (Fig. 3E). And the fluorescence signal in EphA2^−^ SK-MEL-28 cells was significantly attenuated compared to that in 143B and MG63 cells without blocking at the same concentration of YSA-SPION-MV (Fig. 3E). Thus, we concluded that YSA-SPION-MV had significant 143B and MG63 cell targeting ability in vitro, which depended on the binding of YSA peptide to EphA2.

**Fig. 4:**
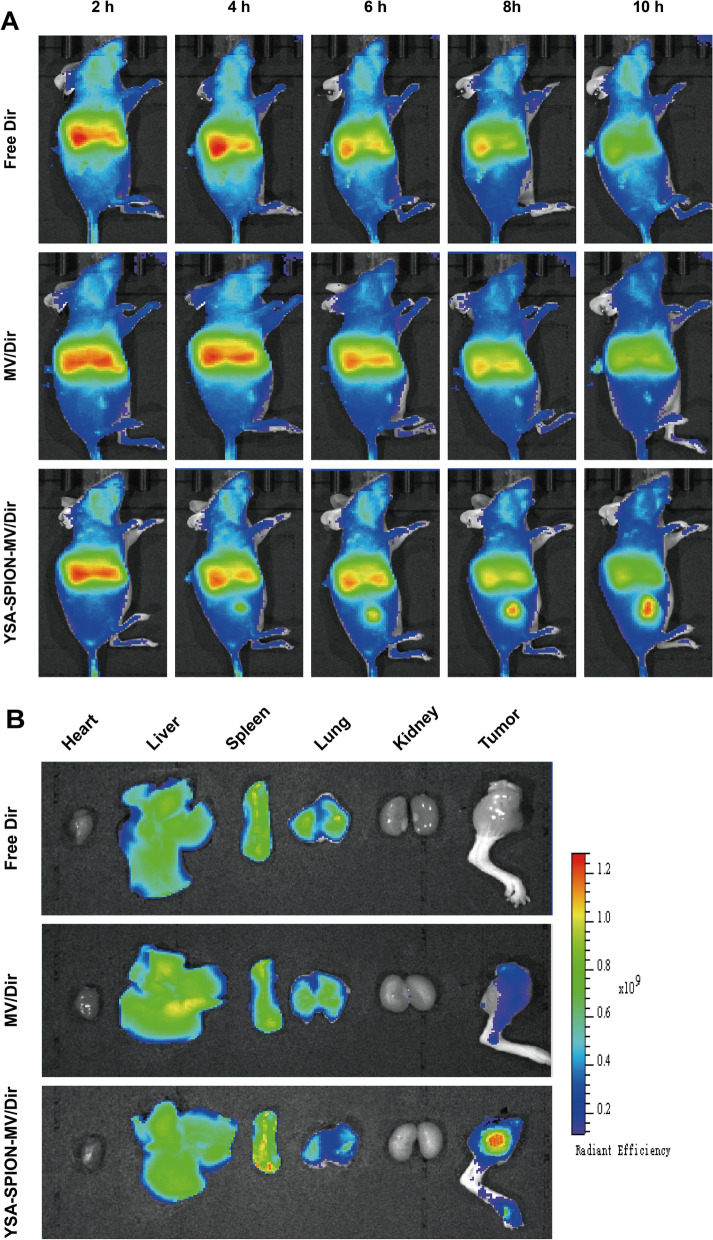
Targeting capability of YSA-SPION-MV in vivo. **A** Fluorescence distribution in tumor-bearing mice after tail vein injection of free Dir, YSA-SPION-MV/Dir or YSA-SPION-MV/Dir. **B** Fluorescence imaging of excised organs from tumor-bearing mice 10 h after tail vein injection of free Dir, YSA-SPION-MV/Dir or YSA-SPION-MV/Dir

Furthermore, the in vivo biodistribution of YSA-SPION-MV/DiR was evaluated using a live imaging system after tail vein injection into the tumor-bearing mice. As shown in Fig. 4A, strong fluorescence was observed in the tumor region of YSA-SPION-MV/Dir-treated mice, while almost no fluorescence was detected in the tumor region of Free Dir-treated mice. The fluorescence of mice treated with MV/Dir was primarily concentrated in the liver and spleen. In contrast, there was a slight fluorescence within the tumor region. Over time, the fluorescence signal in the tumor region of YSA-SPION-MV/Dir-treated mice was observed at 4 h after administration, which increased gradually and was stronger than that in the liver and spleen at 8 h. Consistently, ex vivo imaging of tumors and organs harvested at 10 h (Fig. 4B) revealed that the fluorescence in the tumor of YSA-SPION-MV/Dir group was stronger than that in the other two groups. These results provide a good basis for targeted therapy.

### In vitro anti-tumor effect of YSA-SPION-MV/MTX

**Fig. 5:**
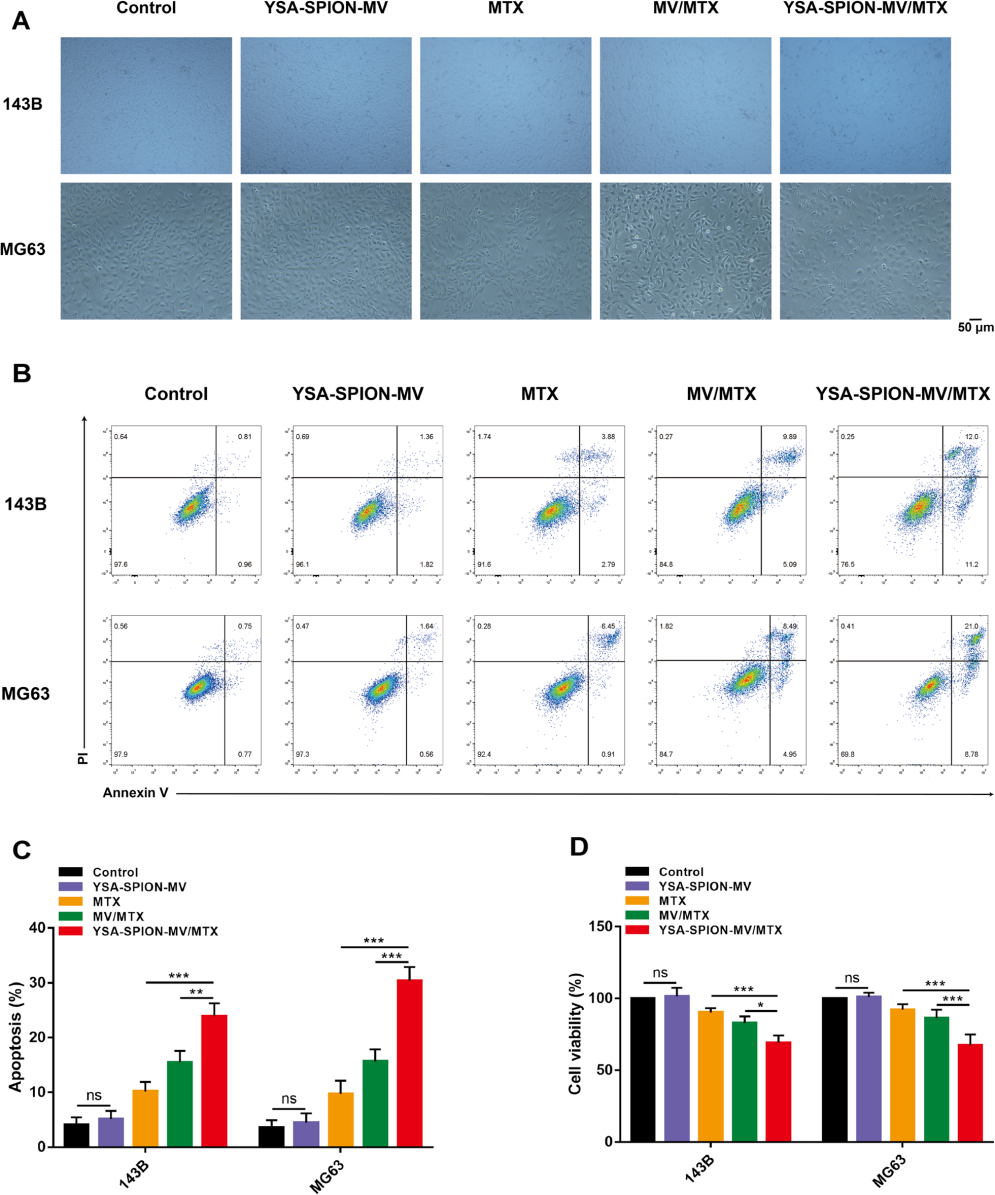
In vitro cytotoxicity of YSA-SPION-MV/MTX. **A** Bright field images of morphology of tumor cells after treatment with PBS (Control), YSA-SPION-MV, MTX, MV/MTX and YSA-SPION-MV/MTX for 24 h. The dose was equivalent to 5 μg/mL MTX (scale bars: 50 μm). **B**, **C** Flow cytometry analysis based on Annexin V-FITC/PI staining of tumor cells after corresponding treatment for 24 h. **D** Relative cell viability of tumor cells was detected by CCK-8 assay after corresponding treatment for 24 h. Ns: no significance, *P < 0.05; **P < 0.01; ***P < 0.001

As shown in Fig. 5A, the cell density of MV/MTX and YSA-SPION-MV/MTX groups decreased significantly, while no apparent change was observed in the MTX group. Meanwhile, the proliferative activity was evaluated quantitatively by CCK-8 and shown in Fig. 5D, no significant decrease was observed after 24-h incubation with YSA-SPION-MV, indicating minimal cytotoxicity of the blank carrier. Compared with the MV/MTX group (143B: 82.81% ± 4.75%; MG63: 86.27% ± 5.92%), the YSA-SPION-MV/MTX group had a lower cell viability rate (143B: 69.10% ± 5.13%, *P* < 0.05; MG63: 67.30% ± 7.60%, *P* < 0.001). The apoptosis of cells was then further examined via flow cytometry (Fig. 5B, C). Consistently, there was no significant difference between the control group and the YSA-SPION-MV group (*P* > 0.05). And YSA-SPION-MV/MTX induced a higher level of cell apoptosis (143B: 23.90% ± 2.36%, *P* < 0.005; MG63: 30.40% ± 2.51%, *P* < 0.001) than MV/MTX (143B: 15.47% ± 2.11%; MG63: 15.63% ± 2.22%).

**Fig. 6:**
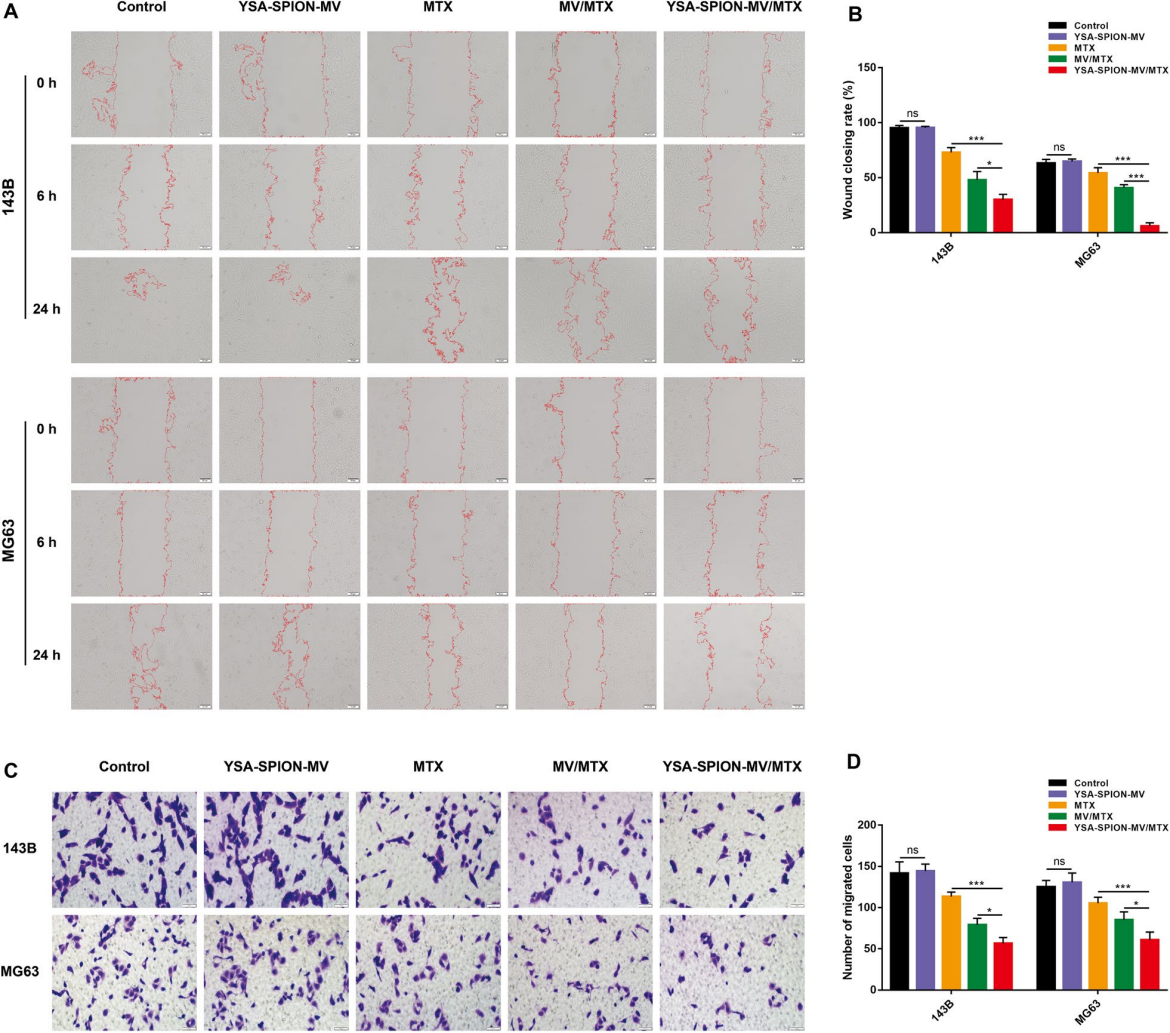
YSA-SPION-MV/MTX inhibited cell migration in vitro*.* The effect of YSA-SPION-MV/MTX on horizontal and vertical cell migration was measured by (**A**, **B**) wound healing assay and (**C**, **D**) transwell assay, respectively. Scale bars: 50 μm. Ns: no significance, *P < 0.05; ***P < 0.001

A wound healing assay as well as a transwell assay were used to evaluate the effects of YSA-SPION-MV/MTX on tumor cell migration. The wound healing assay demonstrated that the scratch width was wider in the MV/MTX group and YSA-SPION-MV/MTX group, but narrower in the MTX group (Fig. 6A). Quantitative analysis suggested that the YSA-SPION-MV/MTX group (143B: 30.06% ± 4.82%, *P* < 0.05; MG63: 5.99% ± 2.96%, *P* < 0.001) significantly decreased the cell migrated area compared to the MV/MTX group (143B: 47.94% ± 7.60%; MG63: 40.70% ± 2.96%) (Fig. 6B). Transwell results (Fig. 6C, D) showed that the number of migrated cells in the YSA-SPION-MV/MTX group (143B: 56.67 ± 7.10, *P* < 0.05; MG63: 60.67 ± 29.61, *P* < 0.001) was significantly lower than that in the MV/MTX group (143B: 79.00 ± 7.94; MG63: 85.00 ± 9.85). Taken together, these results indicated that YSA-SPION-MV/MTX significantly inhibited both horizontal and vertical migration of OS cells in vitro.

### In vivo anti-tumor effect of YSA-SPION-MV/MTX

**Fig. 7:**
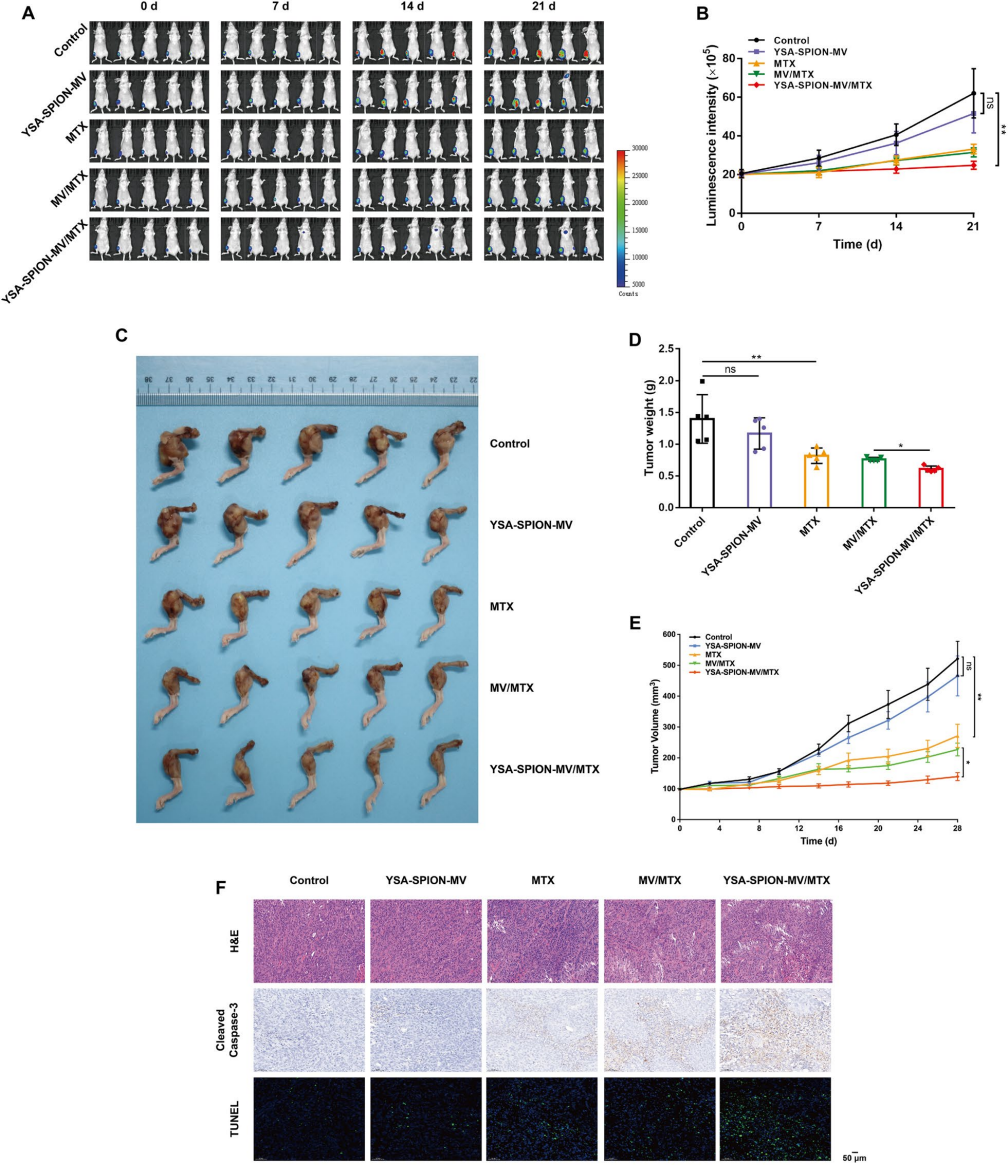
In vivo anti-tumor effect of YSA-SPION-MV/MTX. **A** Bioluminescent images and **B** luminescence intensity quantification of tumor-bearing mice in control, YSA-SPION-MV, MTX, MV/MTX and YSA-SPION-MV/MTX groups (the dose was equivalent to 5 mg/kg MTX). **C** General view of the right hindlimbs from tumor-bearing mice receiving corresponding treatment for 28 days. **D** Tumor weights were measured after corresponding treatment. **E** Tumor volume growth curves of tumor-bearing mice during the administration of corresponding treatment. **F** H&E staining, immunohistochemistry analysis (Cleaved Caspase-3) and TUNEL analysis of tumor in different groups. Scale bars: 50 μm. Ns: no significance, *P < 0.05; **P < 0.01

Inspired by the effective tumor accumulation and satisfying cytotoxicity results of YSA-SPION-MV/MTX in vitro, we further evaluated its in vivo anti-tumor effect in 143B tumor-bearing mice. In vivo bioluminescent imaging was used to monitor tumor growth (Fig. 7A). As shown in Fig. 7B, the luminescence intensity of tumor increased rapidly in the control and YSA-SPION-MV groups, slowly increased in the MTX and MV/MTX groups, but barely in the YSA-SPION-MV/MTX group. On day 21, compared with the control group (62.11 ± 28.46), the luminescence intensity of tumor in the YSA-SPION-MV/MTX group was significantly decreased (24.86 ± 4.61, *P* < 0.005). As revealed by gross observation of tumor-bearing mice (Additional file 5: Fig. S5) and right hindlimbs (Fig. 7C), tumor weights (Fig. 7D) and tumor volumes (Fig. 7E), YSA-SPION-MV/MTX showed the most pronounced tumor growth inhibition. On day 28, compared with the MV/MTX group (227.27 ± 46.24 mm^3^; 0.76 ± 0.03 g), the tumor weight and volume of YSA-SPION-MV/MTX group were significantly reduced (139.40 ± 12.99 mm^3^, *P* < 0.05; 0.61 ± 0.05 g, *P* < 0.05).

To further evaluate the substantial damage to tumor cells by different treatments, H&E, Cleaved Caspase-3, and TUNEL staining were performed (Fig. 7F). In H&E staining, YSA-SPION-MV/MTX caused the most significant large-scale cell shrinkage and severe necrosis. MV/MTX or MTX caused moderate cell necrosis, while no obvious cell damage was found in the YSA-SPION-MV group. Cleaved Caspase-3 and TUNEL staining also indicated that YSA-SPION-MV/MTX caused a significantly higher level of cell apoptosis than MV/MTX and MTX.

**Fig. 8:**
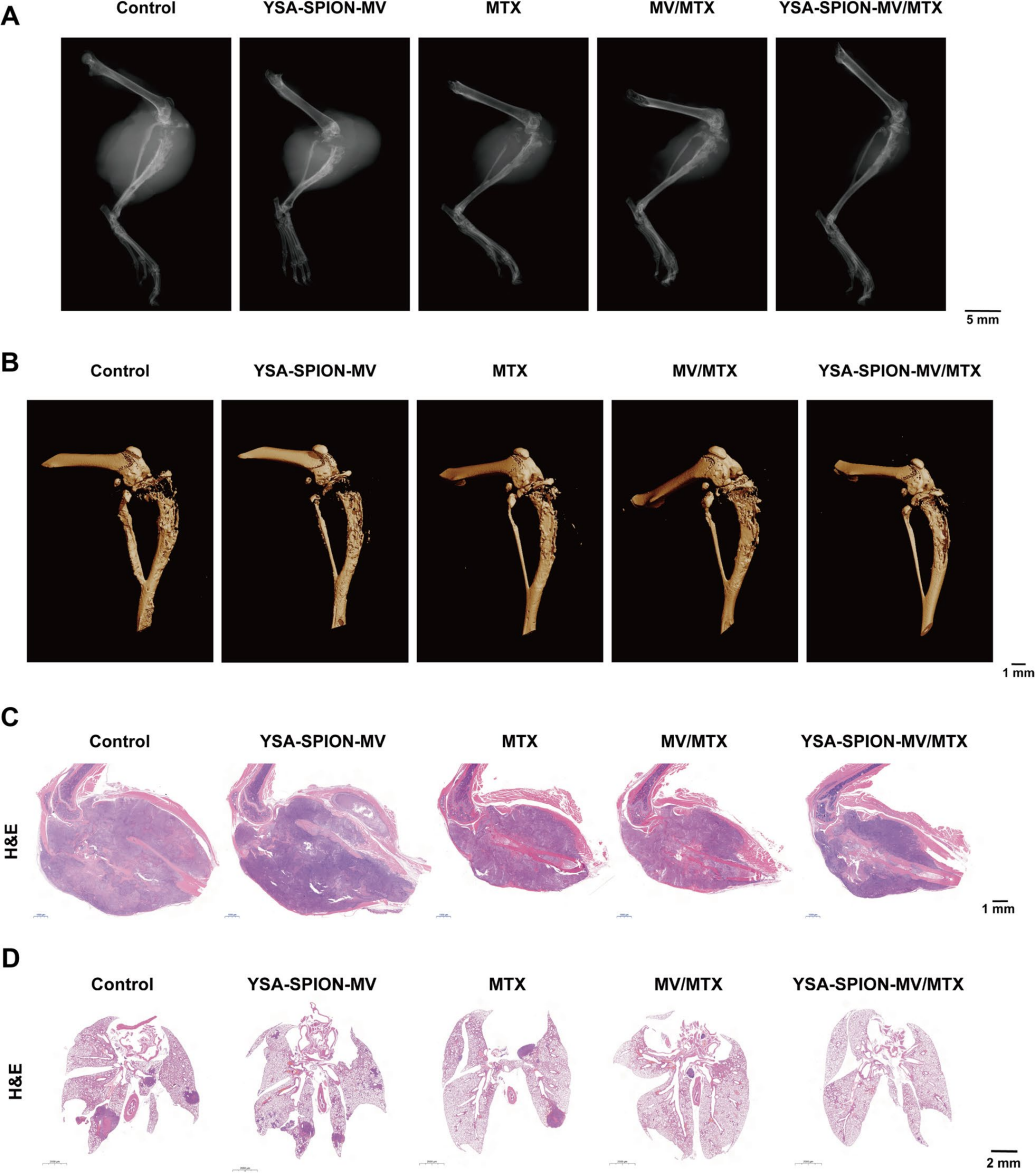
Effect of YSA-SPION-MV/MTX on OS-mediated bone destruction and lung metastasis. Representative **A** X-ray and **B** micro-CT images of right hindlimbs from tumor-bearing mice in control, YSA-SPION-MV, MTX, MV/MTX and YSA-SPION-MV/MTX group (The dose was equivalent to 5 mg/kg MTX) (scale bars: 5 mm and 1 mm). Representative H&E staining images of (**C**) right hindlimbs and (**D**) lung from tumor-bearing mice in different groups (scale bars: 1 mm and 2 mm)

Next, the effect of YSA-SPION-MV/MTX on OS-mediated bone destruction was assessed. As shown in the X-ray and micro-CT images (Fig. 8A, B), pathological fracture was induced by severe osteolysis in the control and YSA-SPION-MV groups. Compared to other groups, treatment with YSA-SPION-MV/MTX reduced bone destruction, especially in the tibial plateau. Consistently, mild osteolysis was also observed in the YSA-SPION-MV/MTX group by H&E staining (Fig. 8C). Meanwhile, the site of bone destruction in the 3D reconstructed image was considered as the region of interest, and then the BMD was analyzed. Unfortunately, the quantitative analysis did not show any statistically significant differences (Additional file 6: Fig. S6A).

In addition, distant metastasis was observed in some tumor-bearing mice according to the bioluminescent imaging (Fig. 7A). To explore this observation further, we conducted H&E staining on the lung, which is the region with the highest probability of distant metastases in OS. The results showed that the number of metastatic lung nodules in the YSA-SPION-MV/MTX group was lower than that in the control, YSA-SPION-MV and MTX groups (Fig. 8D). The lung metastasis area index (*the area of metastatic pulmonary nodules/the area of lung tissue* × *100%*) suggested that YSA-SPION-MV/MTX inhibited the lung metastasis of OS cells in vivo to a certain extent (Additional file 6: Fig. S6B).

### In vivo biosafety evaluation of YSA-SPION-MV/MTX

The toxic effect of drug delivery platforms on major organs and the entire system is a crucial concern in clinical application. As shown in Additional file 7: Fig. S7A, no significant body-weight variations were observed between the control group and the YSA-SPION-MV group. There was a body-weight reduction occurred in the MTX and MV/MTX groups from day 14, which was attributed to the systemic toxicity of MTX. On day 21, compared with the YSA-SPION-MV/MTX group (19.31 ± 1.00 g), the body weight of MV/MTX group was significantly decreased (16.77 ± 0.74 g, *P* < 0.001). Additionally, the H&E staining images of major organs (heart, liver, kidney and intestine) demonstrated that no noticeable tissue damage was observed in the YSA-SPION-MV group (Additional file 7: Fig. S7B). Furthermore, only minor renal tissue damage was observed in the YSA-SPION-MV/MTX group as compared with the MTX and MV/MTX groups. These results collectively certified the favorable biosafety of YSA-SPION-MV/MTX.

## Discussion

The current treatment strategy for osteosarcoma includes neoadjuvant chemotherapy, surgery, and postoperative adjuvant chemotherapy [4, 25]. Despite this aggressive treatment strategy, the 5-year survival rate of OS patients without distant metastases is about 60–70%, compared to only about 20% for OS patients with distant metastases, especially those with lung metastases [4, 5]. High-dose ifosfamide, high-dose methotrexate, cisplatin, and doxorubicin are frequently used in the treatment of osteosarcoma. Due to the lack of targeting ability, chemotherapeutic drugs are commonly accompanied by serious side effects (hepatotoxicity, nephrotoxicity, cardiotoxicity, ototoxicity, etc.) [25, 26]. However, increasing the dose or adding more agents to the treatment regimen does not improve outcomes [25, 27].

Tumor-derived microvesicles (TMVs) have great potential as an emerging drug delivery system for cancer treatment [11]. Previous studies have demonstrated that tumor cells can release a large number of drug-packaging TMVs after co-incubation with chemotherapeutic drugs [28, 29]. As drug delivery vehicles, TMVs can facilitate drug entry into the nucleus of tumor cells and interfere with drug efflux [28, 29]. In addition, drug-packaging TMVs can trigger the formation of a new generation of drug-packaging TMVs, bringing about a domino like tumor killing effect [29]. In this study, we indicated that high drug loading TMVs can be prepared in a straightforward and efficient route.

MVs can carry specific membrane proteins from parent cells; for example, myelin proteins in oligodendrocyte- derived exosomes have the unique property of homing selectivity [30]. Current studies have reported that the tendency of TMVs to target parent cells is one of their representative characteristics [31, 32]. Similarly, we found that there was a slight fluorescence in the tumor region of MV/Dir-treated mice. However, the inherent targeting ability of natural MVs is not ideal, and they tend to accumulate in the mononuclear phagocyte system, leading to rapid clearance. Therefore, targeted surface functionalization of MVs is essential.

In this study, we used YSA peptide-coated SPION to modify the surface of MVs, thereby increasing the recognition and binding ability to EphA2-positive OS cells without affecting the structure of MVs. It is noteworthy that although the percentage of PKH67-positive cells in the MV group was significantly lower than in the YSA-SPION-MV group, it was still around 40%. This suggests that MVs were able to enter cells to a considerable extent, which is plausible since MVs can directly contact with cells in vitro and enter cells through endocytic or plasma membrane fusion effects [33]. Nonetheless, this effect is markedly attenuated if MVs are not derived from the target cells. Qiao et al. reported that the uptake of fibrosarcoma cell line HT108-derived exosomes by HT1080 cells was twice that of HeLa-derived exosomes [31]. Meanwhile, our in vivo biodistribution study showed that unmodified MVs did not accumulate in the tumor region after systemic administration. Under these circumstances, targeting effect is particularly crucial.

Excitingly, the entry of YSA-SPION-MV into cells was significantly reduced when incubated with non-targeted cells or blocked target cells. Thus, we concluded that YSA-SPION-MV had significant EphA2-positive OS cell targeting ability in vitro, and that the targeting ability is dependent on the binding of YSA peptide to EphA2. The difference in cell uptake efficiency under EphA2 receptor blockade or not could be a result of “forward signaling”. As a type of membrane proteins, ephrin triggers a unique bidirectional signaling mechanism: “forward signaling” in the EphA2 receptor expressing cell and “reverse signaling” in the ephrin expressing cell [34, 35]. And the “forward signaling” is usually cell repulsive [36, 37]. Therefore, we speculate that YSA peptide, as a mimetic of ephrinA1, could bind to the EphA2 receptor on the surface of OS cells after co-incubation, thereby initiating “forward signaling” and ultimately reducing the uptake of YSA-SPION-MV.

Recently, biocompatible SPIONs with appropriate structural modifications and conjugated targeting ligands have been widely used for drug delivery applications [20, 38]. Several studies show that SPION-modified EVs have a lower uptake in the liver and spleen, but instead exhibit an increased accumulation at targeted sites such as the spinal cord[39], pancreas [40], and tumor [41]. To deepen our study, we will design a wearable magnetic knee brace to generate a local magnetic field and achieve site‐specific targeting for precision treatment.

## Conclusion

In summary, we designed a therapeutic nanoplatform based on functionalized chemotherapeutic MVs (YSA-SPION-MV/MTX) for targeted osteosarcoma therapy. Our study demonstrated that this nanoplatform had strong OS-specific targeting ability, excellent anti-tumor effect and good biosafety. Several recent clinical trials also highlighted the potential of TMVs as drug carriers for antitumour therapies [42–44]. We hope that our strategy will pave a new path to tailor MV-based therapeutic nanoplatforms for precise cancer treatment and broaden the prospects for basic research and clinical application of MVs.

## Methods

### Materials

Doxorubicin (#HY-N0565) and Methotrexate (#HY-14519) were purchased from MedChemExpress (China). Primary antibodies against TSG101 (#ab125011) and CD63 (#ab134045) were purchased from Abcam (USA). Anti-EphA2 (#6997) and Anti-Cleaved Caspase-3 (#9961) were purchased from Cell Signaling Technology (USA). Surface-carboxyl Fe3O4 superparamagnetic nanoparticles (SPION) was purchased from MukeNano (China). YSAYPDSVPMMS (YSA) was synthesized by GL Biochem (Shanghai) Ltd. (China). PKH67 (#PKH67GL) and Dil (#42364) were purchased from Sigma-Aldrich (USA). Dir (D12731) was purchased from Thermo Fisher Scientific (USA).

### Cell culture and establishment of an orthotopic OS mouse model

The human OS cell lines (143B and MG63) and human melanoma cell line SK-MEL-28 were purchased from the Cell Bank/Stem Cell Bank, Chinese Academy of Sciences. The cells were cultured in Dulbecco’s modified Eagleʼs medium (DMEM) supplemented with 10% fetal bovine serum (FBS). All cells were maintained at 37 °C in a 5% CO_2_ humidified atmosphere.

The orthotopic OS mouse model was established using a reported method [20, 45]. Five-week-old female BALB/c nude mice were anesthetized by intraperitoneal injection of pentobarbital (35 mg/kg). After decontamination, the cortical layer of right tibia was perpendicularly pierced using a 22-gauge needle, followed by insertion of the needle approximately 3–5 mm into the diaphyseal shaft of tibia. After removal of the needle, 5 × 10^7^ 143B-luc cells suspended in 20 μL of PBS were slowly injected with another 22-gauge needle. Gentle pressure was then applied to the injection site to prevent cell leakage. The mice were used for further experiments when the tumor volume reached around 100 mm^3^. The tumor volume was calculated using the following formula: *Tumor volume* = *1/2* × *length* × *(width)*^*2*^. All experimental procedures were approved by the Institutional Animal Care and Use Committee at Tongji Medical College, Huazhong University of Science and Technology.

### Synthesis of functionalized chemotherapeutic MVs

#### Isolation and identification of MVs

Tumor cells were first exposed to ultraviolet irradiation (UVB, 300 J/m^2^) for 1 h. 16 h later, the supernatants were collected for centrifugation: first 10 min at 300*g*, 15 min at 2000*g*, and then 10 min at 14,000*g*. Finally, the supernatants were centrifuged for 90 min at 14,000*g* to pellet the MVs. The pellets were washed three times and resuspended in PBS for the following experiments. For drug loading, chemotherapeutic drug was added to culture medium after UVB exposure.

Total protein of tumor cells and MVs was extracted using RIPA lysis and then centrifuged at 12,000*g*, 4 °C for 30 min. After centrifugation, the total protein concentration of each sample was detected using the BCA kit (Boster Biological Technology, China). Equal amounts of protein from each sample were denatured by boiling in loading buffer, subjected to 10% SDS-PAGE and electroblotted onto PVDF membranes. Following blocking with 5% bovine serum albumin in TBST buffer for 1 h at room temperature, the membranes were incubated with specific primary antibodies overnight at 4 °C. After washing with TBST thrice, the membranes were incubated with secondary antibodies for 1 h at room temperature and washed again. Protein blots were visualized by a Western ECL Substrate Kit (Thermo Fisher Scientific, USA) and a Bio-Rad scanner (Bio-Rad, USA).

MVs were dropped onto the copper grid and incubated for 5 min at room temperature, and then fixed in 5% glutaraldehyde. After removing excess fluid, the samples were negatively stained with 2% phosphotungstic acid at 4 °C and analyzed by transmission electron microscope (TEM, Hitachi, Japan). Besides, the particle size distribution of MVs was measured by dynamic light scattering (DLS; Zetasizer Nano ZS90, Malvern Panalytical, UK).

#### SPION coating with YSA peptide and identification

5 mg 1-Ethyl-3-(3-dimethylaminopropyl)-carbodiimide (EDC) was solubilized in 3 mL 2-(N-morpholino) ethanesulfonic acid (MES) buffer (pH = 7.4, 0.1 mM). 500 μg SPION was solubilized in EDC solution for 15 min, different proportions of YSA peptide and 1 mg N-hydroxysulfosuccinimide (Sulfo-NHS) were added, and then the mixture was shaken overnight at room temperature. The mixture was then transferred into a dialysis bag (MD34, Viskase, USA) and immersed in double-distilled water for 24 h to remove unreacted YSA peptide and catalysts. Finally, YSA-SPION was stored at 4 °C for further experiments.

Fourier transform infrared (FT-IR; Thermo Fisher Scientific, USA) spectroscopy was used to examine the structures of SPION and YSA-SPION. The particle size distribution of SPION and YSA-SPION was measured by DLS. The structure of SPION was observed by TEM.

#### Preparation and identification of YSA-SPION-MV/MTX

MV/MTX was mixed with YSA-SPION (different proportions) overnight at 4 °C to prepare the YSA-SPION-MV/MTX nanocomposite. Unreacted YSA-SPION was removed by ultrafiltration at 3000 rpm for 5 min using 0.2 μm Vivaspin® 6 centrifugal concentrator (Sartorius AG, Germany). YSA-SPION-MV/MTX were resuspended in PBS and stored at − 80 °C for further study. The particle size distribution of YSA-SPION-MV/MTX was measured by DLS and the structure was observed by TEM.

### MV counting

A flow cytometry-based method was used to count the number of MVs as described before [28]. After centrifugation, MVs were suspended in PBS pre-filtered through a 0.1 μm filter, and then passed through a 1 μm filter to further exclude background noise or non-specific events. First, 0.8 μm deep-blue dyed-latex beads (L1398, Sigma-Aldrich, USA) were used for gating and voltage adjustment. Second, the distributions of 0.1 and 3 μm latex beads (LB1 and LB30; Sigma-Aldrich, USA) were analyzed. Finally, MVs were evenly mixed with a known number of LB30 and analyzed by a flow cytometer (BD Biosciences, USA). If 10,000 counts of LB30 were collected, the number of MVs can be calculated with the formula: *N* = *10,000* × *(MV% /LB30%)*.

### Liquid chromatography-tandem mass spectrometry (LC–MS/MS)

MV/MTX was processed by the RIPA lysis buffer for 15 min, and then methanol was added (9 times the volume of buffer). The mixture was vortexed for 2 min and centrifuged at 12,000*g* for 10 min at 4 °C. The supernatant was then evaporated and redissolved with 100 μL 50% methanol, followed by LC–MS/MS analysis. LC was performed using an UltiMate3000RS (Thermo Fisher Scientific, USA). Chromatography was performed on a column (Hypersil Gold, 2.1 × 100 mm, 1.9 μm, Thermo Fisher Scientific, USA) maintained at 30 °C. The gradient elution utilized 0.1% formic acid in water as solvent A and methanol as solvent B at a flow rate of 0.5 mL/min. Tandem mass spectrometry was performed on a TSQ Quantis triple quadrupole mass spectrometer (Thermo Fisher Scientific, USA). The analytes were detected with an electrospray ionization source in the positive ion mode.

### Drug release and stability evaluation

To investigate the in vitro drug release pattern of MTX from YSA-SPION-MV/MTX, 2 mL YSA-SPION-MV/MTX was transferred into a dialysis bag immersed in pH7.4 and pH5.5 PBS maintained at 37 °C under 100 rpm shaking. At the specified time points (2, 4, 8, 16, 24, and 48 h), the amount of MTX in the dialysis bag was measured by UV–vis spectroscopy at λmax = 305 nm [46].

Stability of YSA-SPION-MV/MTX was evaluated by monitoring the gross characters (clarity, color, and solubility), morphology, particle size and concentration of SPION. Ultrafiltration was used to remove potential YSA-SPION separated from MV/MTX, and the content of SPIONs in YSA-SPION-MV/MTX was measured by UV–vis spectroscopy at λmax = 510 nm.

### *Cell uptake and targeting capability *in vitro

143B or MG63 cells were seeded in 6-well plates (1 × 10^5^ cells per well) and cultured overnight. The cells were incubated with PKH67-labelled MV and YSA-SPION-MV (MV/cell = 5:1) for 6 h or 12 h, respectively. Cells without any treatment were used as control. After incubation, cells were trypsinized, resuspended in PBS, and then analyzed on a flow cytometer for quantitative analysis.

For the targeting study, EphA2-positive 143B and MG63, and -negative SK-MEL-28 cells, which were pre-stained with Dil, were treated with PKH67-labelled YSA-SPION-MV. After incubation for 6 h, the cells were fixed and observed using a confocal microscope (Olympus Optical Co., Ltd, Japan). Meanwhile, for competitive inhibition experiments, EphA2^+^ 143B or MG63 cells were pre-incubated with YSA peptide (5 μg/mL) at 37 °C for 30 min before the PKH67-labelled YSA-SPION-MV was added. Finally, the cells were fixed and observed using a confocal microscopy.

### In vivo* biodistribution analysis*

Free Dir, MV/Dir and YSA-SPION-MV/Dir were injected into the tumor-bearing mice via tail vein at a single Dir dose of 50 μg/kg. At the designated time points (2, 4, 6, 8 and 10 h), mice were anesthetized and imaged using the IVIS small animal imaging system (PerkinElmer Inc., USA). At the final time point, mice were sacrificed and major organs (heart, liver, spleen, lung and kidney) as well as tumors were harvested and imaged immediately.

### Cell viability and cell apoptosis assay

Cell viability was determined by using the cell counting kit-8 (CCK-8 Boster, Biological Technology, China) assay. In brief, 143B or MG63 cells were seeded in 96-well plates (5000 cells per well) and cultured overnight. After the corresponding treatment, cells were cultured for 24 h. The supernatant was then discarded and washed with PBS. Afterwards, the CCK8 reagent was then added to the plates. After incubation in dark for 2 h, the absorbance value at A450 nm was detected using a microplate reader (BioTek, USA).

143B or MG63 cells were seeded into 6 well plates and treated correspondingly for 24 h. The cells were then trypsinized, washed with PBS, stained with Annexin V-FITC and propidium iodide (PI, BD Biosciences, USA), and analyzed using a flow cytometer.

### Wound healing assay and transwell assay

143B or MG63 cells were seeded in 6-well plates and cultured to confluence. The cell monolayer was artificially scratched with a 10 μL pipette tip. Then the plate was washed with PBS and the culture medium was replaced by serum-free DMEM. After the corresponding treatment, the wound areas were photographed under an inverted microscope at 0, 6 and 24 h.

A transwell chamber (8-µm pore size, Corning-Costar, USA) was used for transwell assays. Briefly, after the corresponding treatment, 5 × 10^4^ 143B or MG-63 cells in 200 µL serum-free DMEM were seeded in the upper chamber. The lower chamber was flooded with 500 µL 10% FBS DMEM. After incubation for 24 h, cells that migrated to the lower chamber were fixed with 4% polyoxymethylene and stained with 0.05% crystal violet. The migrated cells were imaged and counted under an inverted microscope.

### Animal study

Tumor-bearing mice were equally randomized into five groups, and administered with PBS (control), YSA-SPION-MV, MTX, MV/MTX or YSA-SPION-MV/MTX twice per week, respectively. The dose was equivalent to 5 mg/kg MTX. Body weight and tumor volume were monitored on day 0, 3, 7, 10, 14, 17, 21, 25 and 28. For in vivo bioluminescence imaging, the luciferase substrate D-Luciferin was injected prior to imaging on day 0, 7, 14 and 21. On day 28, the mice were sacrificed and major organs (lung, heart, liver, kidney and intestine) as well as tumors were harvested and fixed with 4% paraformaldehyde.

The destruction of tibia was assessed by X-ray and micro-computed tomography (micro-CT, Scanco Medical, Switzerland). CT images were taken at a resolution of 35 μm (achieved using 65 kV and 190 μA). The bone mineral density (BMD) was measured.

To evaluate the therapeutic efficacy, tumor tissue was examined by hematoxylin and eosin (H&E) staining, immunohistochemistry analysis (Cleaved Caspase-3) and terminal deoxynucleotidyl transferase-mediated dUTP nick end labeling (TUNEL) assay. To assess the in vivo biosafety of the treatments, major organs (heart, liver, kidney and intestine) were examined by H&E staining. Besides, lung was also examined by H&E staining to evaluate lung metastasis. Renal lesions were graded as follows (scores 0–4). 0 = Normal histology. 1 = Focal tubular cell degeneration and intratubular granular debris. No significant necrosis/apoptosis and epithelial cell desquamation. 2 = Tubular epithelial necrosis/apoptosis and epithelial cell desquamation in less than 25% of the tubules, and accompanied by other concomitant alterations. 3 = Tubular epithelial necrosis/apoptosis and epithelial cell desquamation in 25–75%, and accompanied by other concomitant alterations. 4 = Tubular epithelial necrosis/apoptosis and epithelial cell desquamation in more than 75%, and accompanied by other concomitant alterations [47].

### Statistical analysis

All experiments were performed at least three times and results were expressed as mean ± SD. All data were analyzed by GraphPad Prism v.8.4.0 software (GraphPad, USA). The differences between any two groups were determined by the student’s t-test. One-way analysis of variance (ANOVA) followed by Tukey’s test was used to compare the differences among three or more groups. A *P* value < 0.05 was considered to show statistically significant differences.

### Supplementary Information

**Additional file 1: Fig. S1**. The distributions of 0.1, 0.8 and 3 μm latex beads, PBS, mixed latex beads, mixture of 3 μm latex beads and MVs were analyzed by flow cytometry.

**Additional file 2: Fig. S2**. HPLC analysis of the amount of MTX in MVs.

**Additional file 3: Fig. S3**. General view of different proportions of (A) YSA-SPION and (B) YSA-SPION-MV.

**Additional file 4: Fig. S4**. Drug release and stability of YSA-SPION-MV/MTX. (A) MTX release of YSA-SPION-MV/MTX for 48 h at pH7.4 and pH5.5 at 37 °C. (B) The relative content of SPIONs in YSA-SPION-MV/MTX during 48 h at pH7.4 at 4 °C. The gross characters (C), particle size (D) and morphology (E) of YSA-SPION-MV/MTX after 2 weeks at pH7.4 at -80 °C. Scale bars: 200 nm.

**Additional file 5: Fig. S5**. General view of tumor-bearing mice after corresponding treatment.

**Additional file 6: Fig. S6**. Quantitative analysis of (A) BMD in the region of interest and (B) lung metastasis area index.

**Additional file 7: Fig. S7**. In vivo biosafety evaluation of YSA-SPION-MV/MTX. (A) Body weight changes of mice in each group during treatment. (B) Representative H&E staining images of each major organ from tumor-bearing mice in different groups. (C) Quantitative analysis of renal histopathological score. Scale bars: 50 μm. Ns: no significance, **P < 0.01; ***P < 0.001.

## Data Availability

The data are available from the corresponding author on reasonable request.
