# Supplementary figures and images for: EphA2-specific microvesicles derived from tumor cells facilitate the targeted delivery of chemotherapeutic drugs for osteosarcoma therapy
Source: J Nanobiotechnology. 2024 Mar 3;22:89. doi: 10.1186/s12951-024-02372-0 (PMC10909271; doi:10.1186/s12951-024-02372-0)

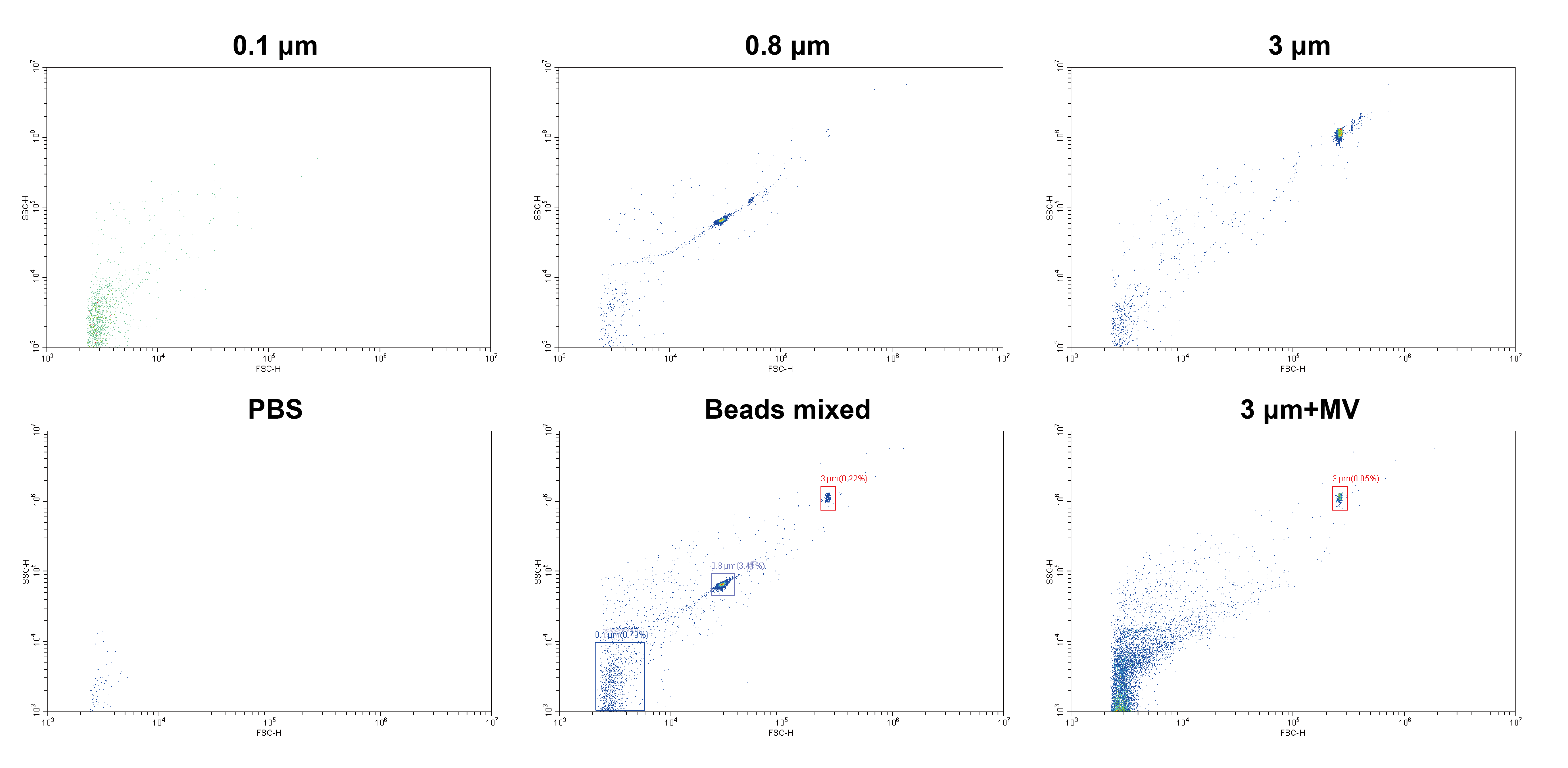

Supplement: Supplementary file 1 — Additional file 1: Fig. S1. The distributions of 0.1, 0.8 and 3 μm latex beads, PBS, mixed latex beads, mixture of 3 μm latex beads and MVs were analyzed by flow cytometry. [file 12951_2024_2372_MOESM1_ESM.png]

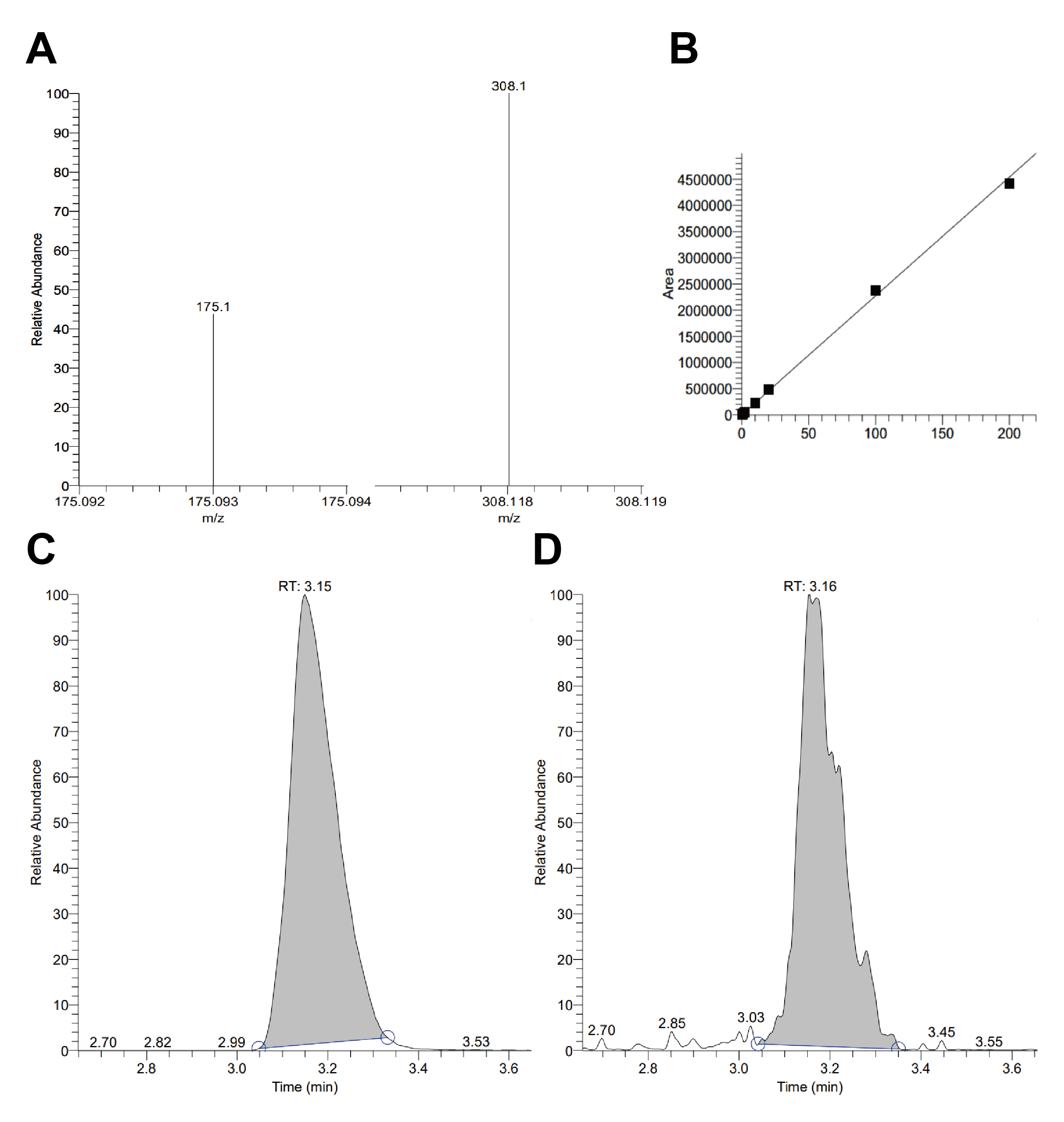

Supplement: Supplementary file 2 — Additional file 2: Fig. S2. HPLC analysis of the amount of MTX in MVs. [file 12951_2024_2372_MOESM2_ESM.png]

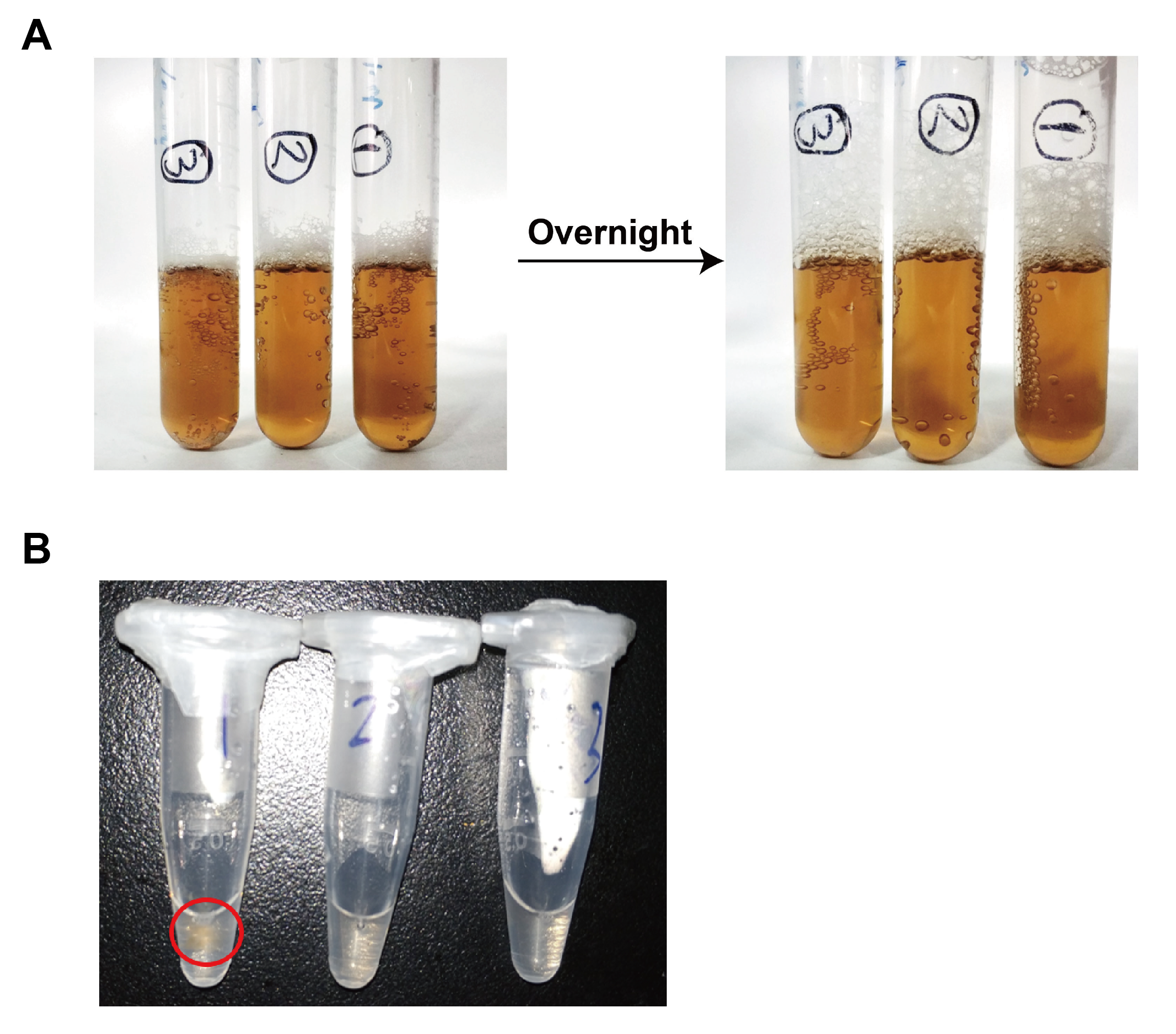

Supplement: Supplementary file 3 — Additional file 3: Fig. S3. General view of different proportions of (A) YSA-SPION and (B) YSA-SPION-MV. [file 12951_2024_2372_MOESM3_ESM.png]

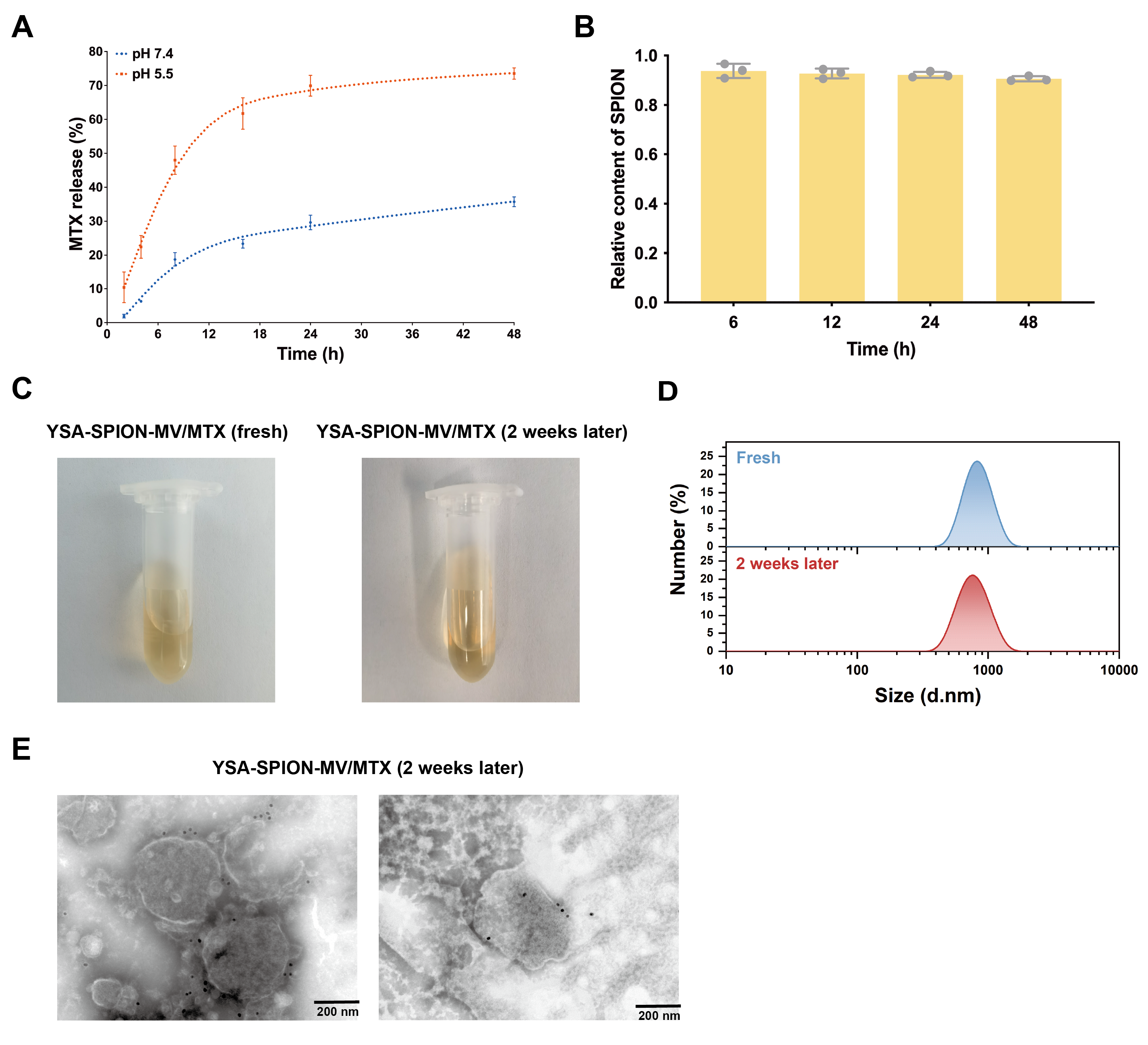

Supplement: Supplementary file 4 — Additional file 4: Fig. S4. Drug release and stability of YSA-SPION-MV/MTX. (A) MTX release of YSA-SPION-MV/MTX for 48 h at pH7.4 and pH5.5 at 37 °C. (B) The relative content of SPIONs in YSA-SPION-MV/MTX during 48 h at pH7.4 at 4 °C. The gross characters (C), particle size (D) and morphology (E) of YSA-SPION-MV/MTX after 2 weeks at pH7.4 at -80 °C. Scale bars: 200 nm. [file 12951_2024_2372_MOESM4_ESM.png]

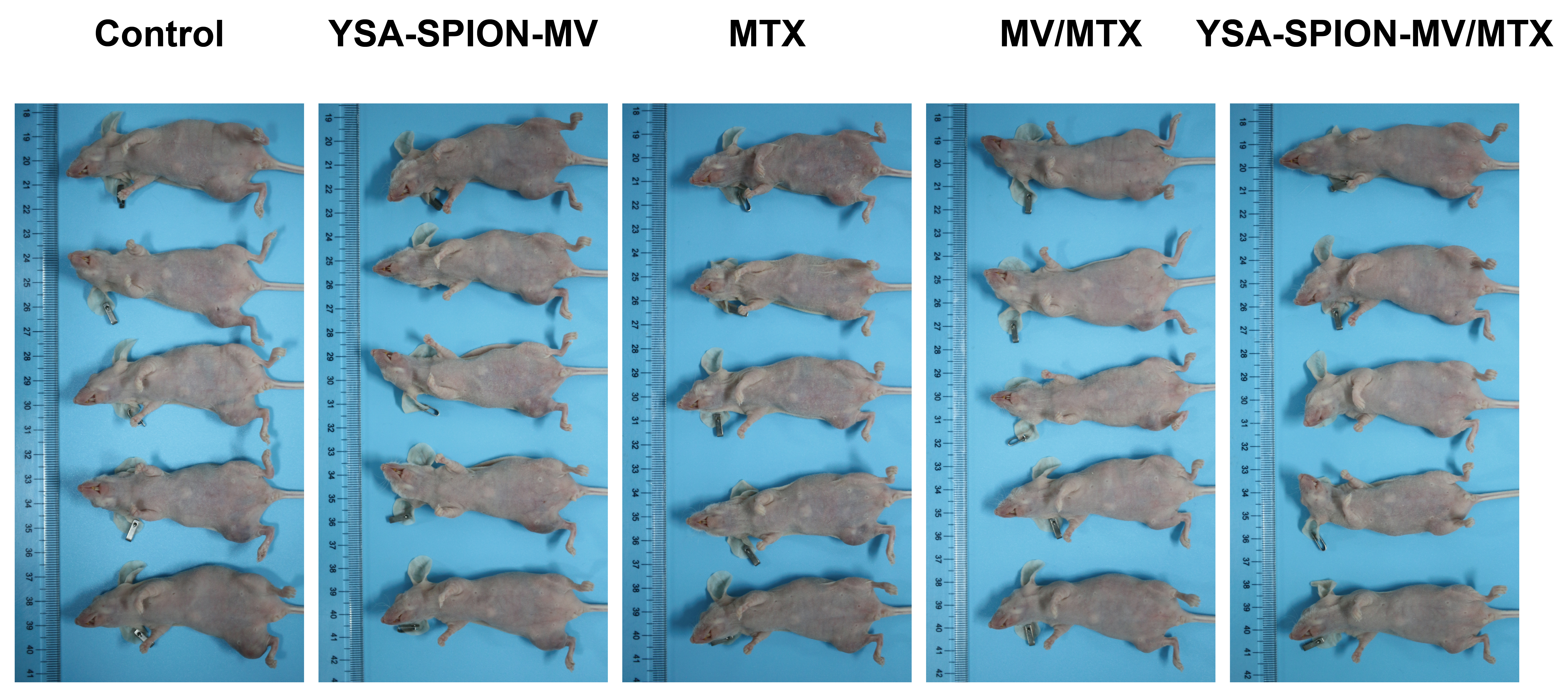

Supplement: Supplementary file 5 — Additional file 5: Fig. S5. General view of tumor-bearing mice after corresponding treatment. [file 12951_2024_2372_MOESM5_ESM.jpg]

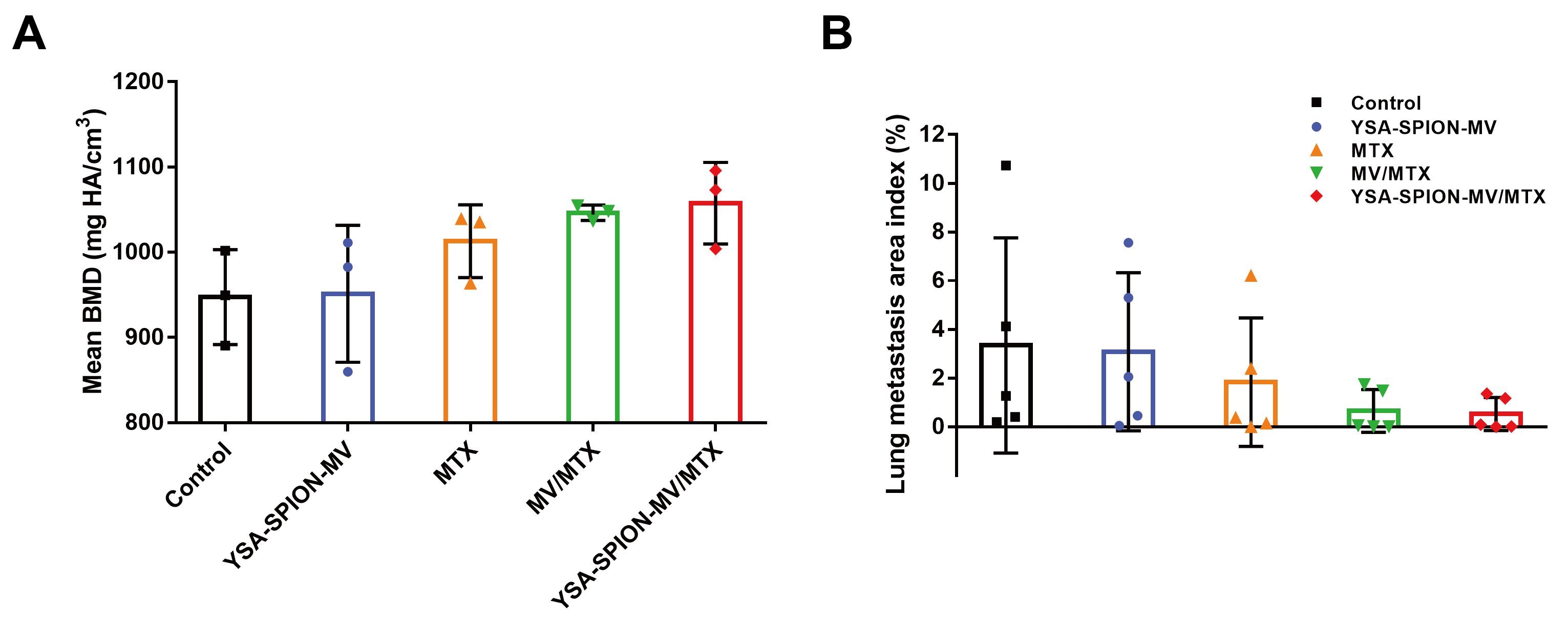

Supplement: Supplementary file 6 — Additional file 6: Fig. S6. Quantitative analysis of (A) BMD in the region of interest and (B) lung metastasis area index. [file 12951_2024_2372_MOESM6_ESM.png]

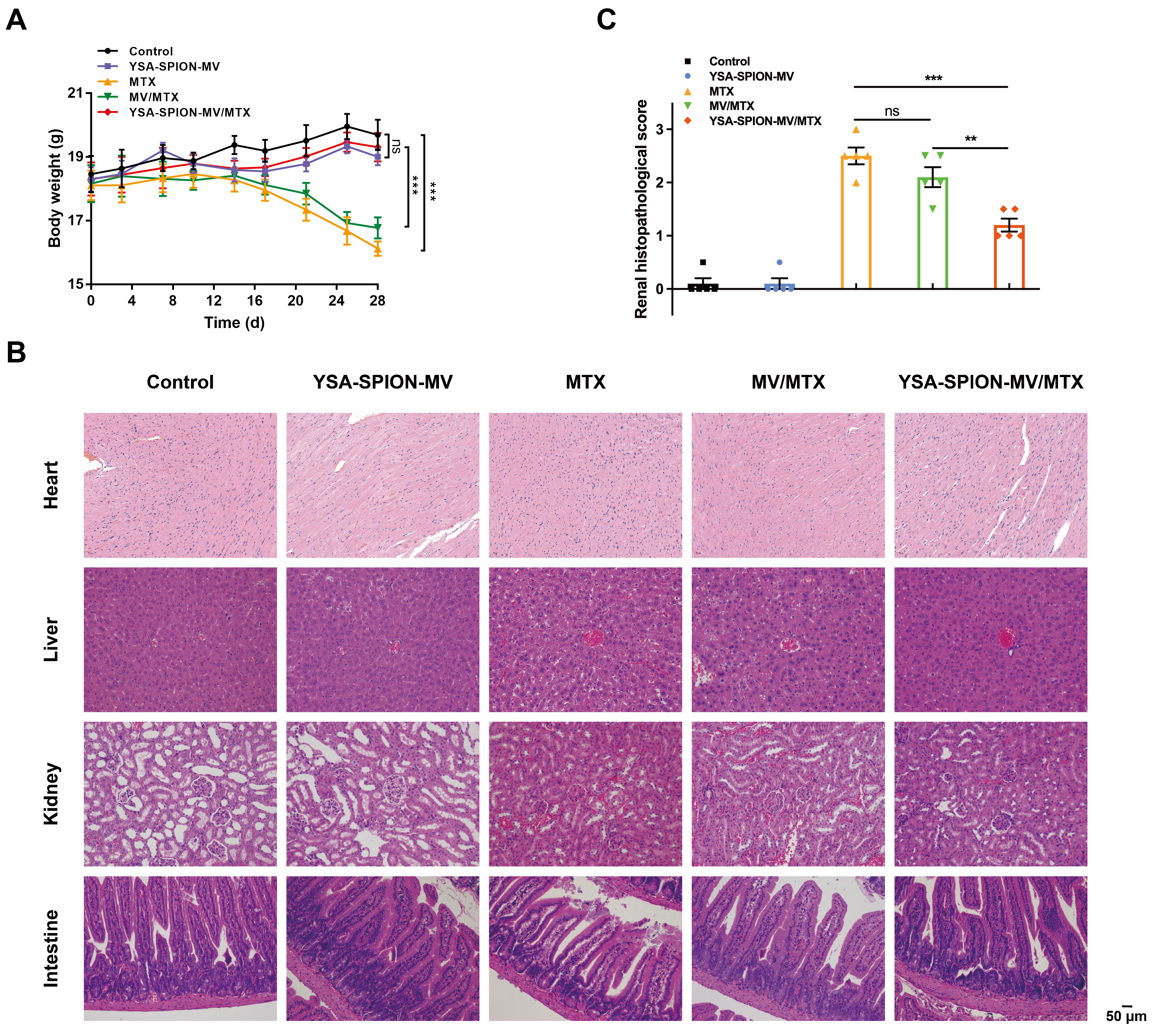

Supplement: Supplementary file 7 — Additional file 7: Fig. S7. In vivo biosafety evaluation of YSA-SPION-MV/MTX. (A) Body weight changes of mice in each group during treatment. (B) Representative H&E staining images of each major organ from tumor-bearing mice in different groups. (C) Quantitative analysis of renal histopathological score. Scale bars: 50 μm. Ns: no significance, **P < 0.01; ***P < 0.001. [file 12951_2024_2372_MOESM7_ESM.jpg]
